# Three-dimensional evaluation of alveolar bone and pharyngeal airway dimensions after mandibular dentition distalization in patients with Class III malocclusion: a retrospective study

**DOI:** 10.1186/s13005-023-00382-1

**Published:** 2023-08-30

**Authors:** Zhijie Zhou, Liangyan Sun, Fan Zhang, Yan Xu

**Affiliations:** 1 Department of Orthodontics, Shanghai Stomatological Hospital & School of Stomotology, Fudan University, Shanghai, China; 2 Shanghai Key Laboratory of Craniomaxillofacial Development and Diseases, Fudan University, Shanghai, China

**Keywords:** Alveolar bone, Pharngeal airway dimensions, Orthodontic treatment, Three-dimensional measurements, Cone-beam computed tomography (CBCT)

## Abstract

**Background:**

To three-dimensionally evaluate changes of the alveolar bone around the mandibular anterior teeth and pharyngeal airway dimensions in adults with Class III malocclusion before and after orthodontic treatment of mandibular dentition distalization.

**Methods:**

In this retrospective study, cone-beam computed tomography (CBCT) scans of 20 patients with Class III malocclusion who underwent mandibular dentition distalization were obtained both before and after treatment. Three-dimensional changes of the thickness and vertical marginal bone levels around mandibular incisors and canines were assessed and compared. And airway volumes of the palato-, glosso-, laryngopharynx and the minimum axial area were measured and compared before and after treatment.

**Results:**

A significant decrease of lingual bone thickness of mandibular incisors, partial labial and lingual bone thickness of canines were observed (P < 0.05). The reduction in root length of incisors and canines, labial and lingual vertical marginal bone levels were significant after orthodontic treatment. No significant correlations between mandibular dentition distalization and pharyngeal airway dimensions were observed.

**Conclusions:**

Mandibular dentition distalization could result in the loss of alveolar bone around anterior teeth in Class III malocclusion, especially for the cervical marginal bone. Pharyngeal airway dimensions were not affected to a high extent after distalization.

**Trial registration:**

Retrospctively registered.

## Background

The orthodontic treatment of Class III malocclusion in adults can be briefly summarized as teeth extraction, distalization of mandibular dentition, and combined orthodontic-orthognathic treatment for improving mandibular protrusion and establishing normal overjet. Specific treatment methods are influenced by patterns of growth, arch crowding, severity of skeletal malocclusion, and patient aesthetic requirements [1]. Compared to the total arch retraction of the upper dentition, distalization of lower dentition is more difficult owing to the anatomical limitations of mandible, including the lingual cortex and posterior available space for distalization [2]. However, in the correction of mild-to-moderate Class III malocclusion, mandibular dentition distalization can provide effective outcomes like a favorable profile change and a stable occlusion [3]. Hence, in some borderline cases, mandibular dentition distalization is widely accepted by patients who are unwilling to extract more teeth [4].

The approaches of distal movement are made up of extraoral appliances (mandibular headgear), intraoral devices, including lip bumper, pendulum appliances, nickel-titanium open coil springs, and mini-screws [5]. Mini-screws are more acceptable to patients due to their comfort, less patient compliance requirements, and visible effects. On the other hand, their flexible insertion location, mechanical resistance and the absolute anchorage made mandibular dentition distalization simple but efficient [5, 6]. However, there are limits to sagittal tooth movement. Fenestration, bone dehiscence, and gingival recession would occur after orthodontic treatment, if excessive anterior teeth movement is performed [7]. These side effects were investigated by some authors both 2-dimensionally and 3-dimentionally [8, 9]. Sun Q [10] evaluated the alveolar bone width and height in both maxillary and mandible area after extraction of two premolars using lateral cephalograms. The results showed that the alveolar bone width decreased significantly in both maxilla and mandible, except on the labial side of mandible. Valerio [7] investigated that bone dehiscence developed on the labial side of mandibular incisors after the treatment crowding without extraction through CBCT. Few studies have focused on periodontal lesions during the overall distal movement of the lower dentition without extraction, let alone the pharyngeal airway changes after orthodontic treatment.

The upper airway is made up of the nasopharynx, oropharynx and laryngopharynx, of which the oropharynx is the narrowest part. And it is susceptible to changes after orthodontic treatment [11]. It has been reported that not only the tongue, soft palate and pharyngeal fat pads but also the positions of maxilla and mandible would influence the airway volume [12]. Many researchers have focused on the influence of premolar extractions on the upper airway. Karaman [13] found that the orthodontic treatment of premolar teeth extraction did not affect pharyngeal airway dimensions significantly by radiograph analyses. However, another study indicated that premolar teeth retraction would lead to the reduction of the airway volume [14]. Changes in the upper airway dimensions after orthodontic treatment of mandibular dentition distalization have received little attention.

Therefore, the aim of this study was to evaluate changes of the alveolar bone around the mandibular anterior teeth and pharyngeal airway dimensions in adults with Class III malocclusion before and after orthodontic treatment of mandibular dentition distalization in three dimensions, thereby providing a theoretical basis for periodontal assisted orthodontic treatment.

## Materials and methods

### Subject selection

The subjects were retrospectively collected from the files of Shanghai Stomatological Hospital and School of Stomatology, Fudan University. The cone-beam computed tomography (CBCT) scans of 20 patients with Class III malocclusion who had undergone orthodontic treatment of distal movement of lower dentition were obtained both before (T0) and after (T1) treatment. The patients consisted of 5 males and 15 females (mean age, 27.5 ± 7.22 years). The specific inclusion criteria were as follows: (a) adult patients with mild class III malocclusion, ANB > -2° and the lower dentition crowding ≤ 4 mm; (b) complete mandibular dentition, i.e. 7–7 complete; (c) straight arch wire technique were applied and micro implants were used in distal movement of lower dentition; (d) all patients had completed orthodontic treatment, and CBCT was taken before and after orthodontic treatment; (e) there was no orthodontic intervention before taking CBCT; (g) no systemic history or mental illness.

The exclusion criteria were as follows: (a) mandible with cyst, fracture and tumor; (b) patients with severe periodontitis; (c) patients with maxillofacial trauma history, temporomandibular joint ankylosis or osteopathy, craniomaxillofacial malformation syndrome, severe maxillofacial asymmetry; (d) CBCT images were too vague to measure or the mandibular morphology was incomplete.

### CBCT data analysis

CBCT images were taken by skilled operators at the beginning and ending of the orthodontic treatment with 3D Accuitomo—XYZ Slice View Tomograph (Morita Dental Company, Japan) under the following conditions: 90 kV, 6.0 mA, 17.5 s of scan duration and a 170 * 120 mm field of view selection with a basic voxel size of 0.125 mm. Patients were asked to put on radiation protective clothing. The head of the patient was in its natural position, and the Frankfort plane was parallel to the floor. The teeth were in the maximal intercuspation. A forehead strap and chin rest were used to fix the head position. The patients were asked not to swallow during the scan for more precise assessment of pharyngeal airway dimensions. Skilled operators were systematically trained in the procedures of CBCT operations. Weekly spot checks were performed on operators and on CBCT images to maintain the consistent CBCT imaging parameters. Unqualified images would be screened out again.

All scan data were reformatted and processed by Dolphin imaging (Version 11.9.07.24 Premium), InVivoDental Application (Version 5.3.5) and OnDemand3D Application (Version 2.0.10.9499). The lateral cephalometric radiographs were obtained using Dolphin imaging, followed by digital cephalometric analysis and pharyngeal airway analysis. OnDemand3D Application was used to measure the root length of mandibular incisors and canines, the alveolar bone thickness on both labial and lingual sides and the vertical marginal bone level. Besides, the black triangle areas were measured by InVivoDental Application.

With Dolphin imaging, the lateral cephalometric radiographs were obtained both left and right orientation for measuring the angle between the mandibular first molar (L6) and the mandible plane, as well as the angle between the mandibular second molar (L7) and the mandible plane. Skeletal variables also included SNA, SNB, ANB, Wits, IMPA, MP-OP and OP-FH.

**Fig. 1:**
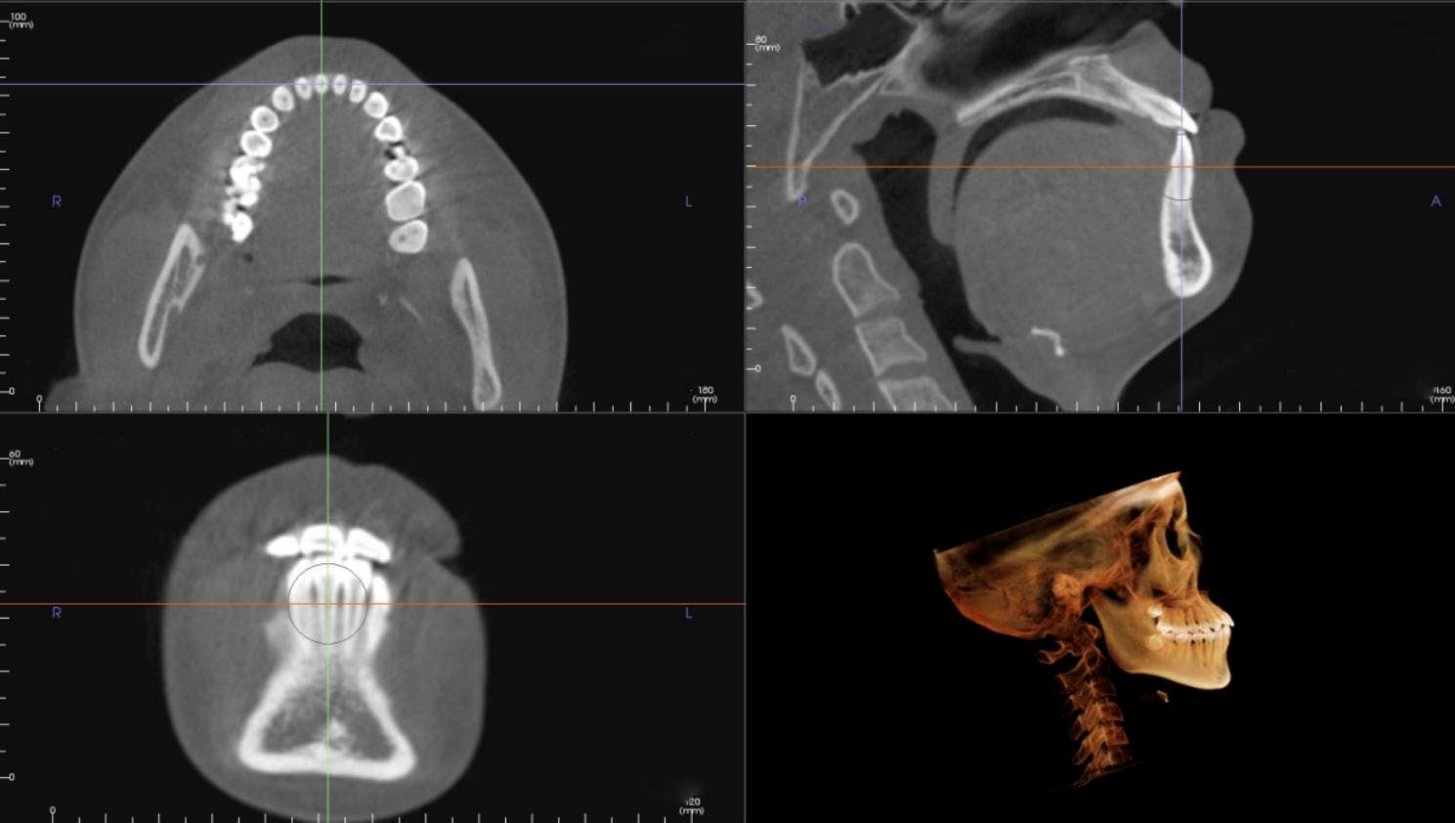
The reference plane was adjusted to pass through the axis of the examined teeth, while the sagittal plane was perpendicular to the labial surface of teeth and it passed through the cementoenamel junction (CEJ).

**Fig. 2:**
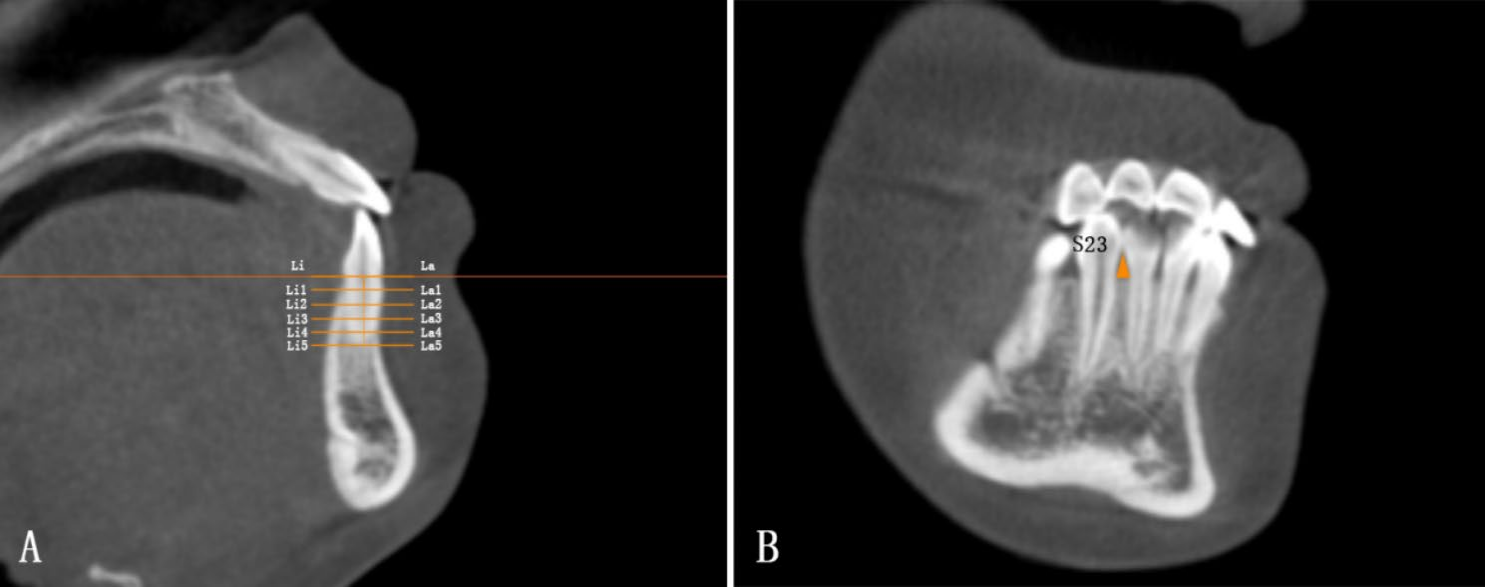
**A**: Measurements of the thickness of the alveolar bone both on the labial side and lingual side. The root length was evenly devided into five levels. La and Li represented CEJ, while La5 and Li5 represented root apex. La1-La5 and Li1-Li5 meant to be the distance between the cortical plate and the root surface on the labial side and lingual side respectively. **B**: Black triangle area between the lateral incisor and canine

Using OnDemand3D Application and InVivoDental Application, the measuring plane was identical to the previous study [15]. It was adjusted to pass through the axis of the examined teeth, while the sagittal plane was perpendicular to the labial surface of teeth. The tooth axis was the line between the midpoint of the incisor edge and the point of root apex (Fig. 1). The root length of mandibular incisors and canines were measured, which was defined as the distance from the cementoenamel junction (CEJ) to root apex. Before treatment, the root length was evenly divided into five levels. La and Li represented CEJ, while La5 and Li5 represented root apex. And after treatment, the interval continued to be used accordingly in measuring the thickness of alveolar bone, which meant to be the distance between the cortical plate and the root surface (Fig. 2). Vertical marginal bone level (VBL) was the distance between alveolar marginal bone crest (AC) and the CEJ on both labial and lingual sides. As to the area of black triangle between the central and lateral incisor, it was defined as the area enclosed by the distal AC of central incisor, the mesial AC of lateral incisor and the approximal point of them, which was similar to the area of black triangle between the lateral incisor and canine (Fig. 2).

**Fig. 3:**
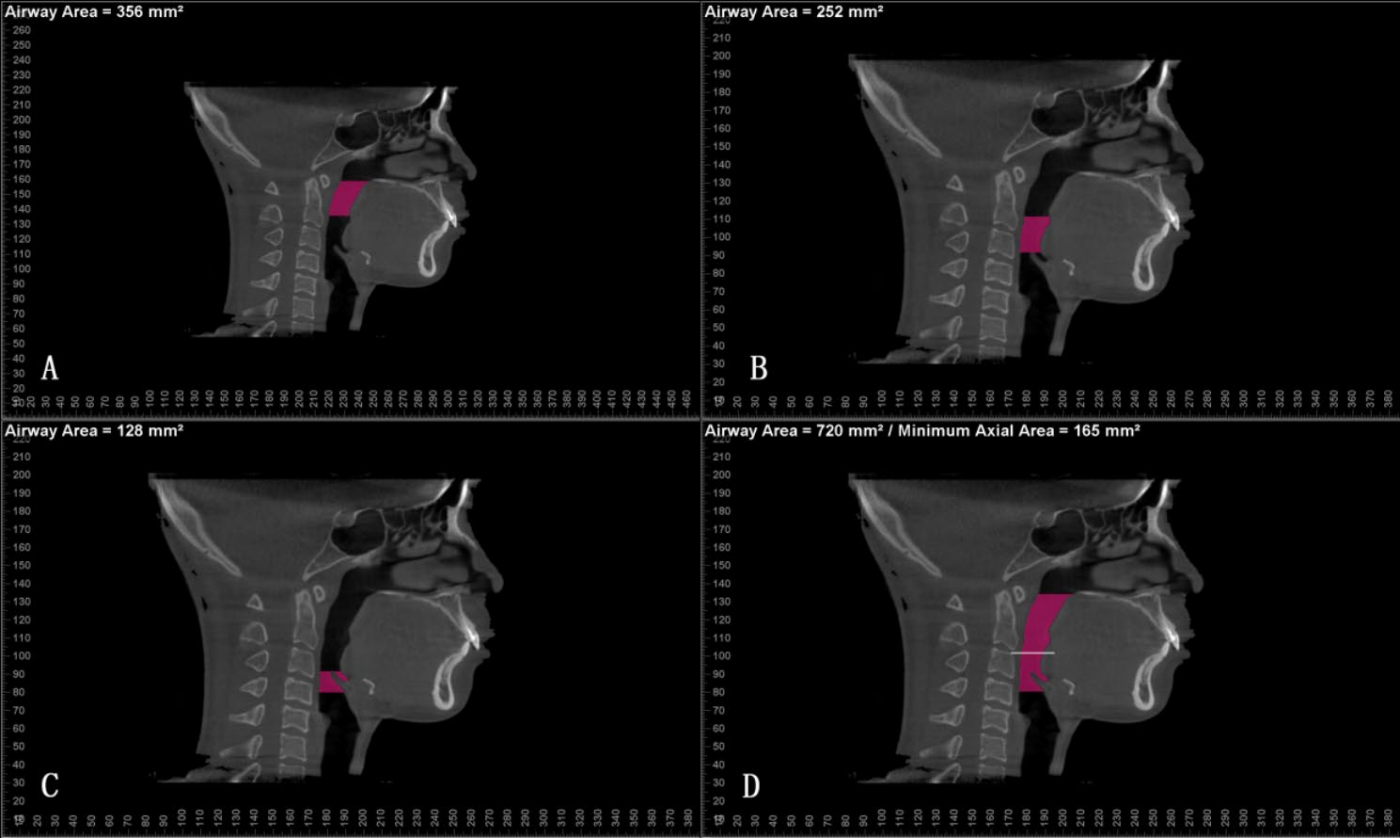
**A**: Palatopharyngeal airway: defined as the space between the planes parallel to the FH plane through PNS and through uvula point (U point). **B**: Glossopharyngeal airway: defined as the space between the planes parallel to the FH plane through U point and epiglottis point (E point). **C**: Laryngopharyngeal airway: defined as the space between the planes parallel to the FH plane through E point and basal part of the epiglottis (BE point). **D**: The minimum axial area of the pharyngeal airway

The pharyngeal airway was divided into three parts according to the tip of the uvula (U point) and the apex point of the epiglottis (E point) [16]. The sinus/airway module in Dolphin imaging was utilized to segment the airway. The sensitivity was adjusted to be 50 in order to limit the range and accuracy. The palatopharyngeal airway was defined as the space between the planes parallel to the FH plane through PNS and through U point. The glossopharyngeal airway was defined as the space between the planes parallel to the FH plane through U point and E point. Laryngopharyngeal airway was defined as the space between the planes parallel to the FH plane through E point and basal part of the epiglottis (BE point). The total upper pharyngeal airway was defined as the space between the planes parallel to the FH plane and BE point. Then, the volume of each space was automatically calculated. Besides, the minimum axial area of the pharyngeal airway was identified and calculated automatically (Fig. 3).

### Statistical analysis

IBM SPSS Statistics (Statistica for Windows, version 24.0) was used for statistical analysis at the 5% level of significance. Before comparing the linear measurements at T0 and T1, a normality test was performed. The Shapiro-Wilk test showed a normal distribution of the measurements. The lateral cephalometric variables, alveolar bone thickness of central incisors and canines, root length and vertical marginal bone level were compared by the paired t-test between T0 and T1. Additionally, the pharyngeal airway volumes and minimum axial area were analyzed. A p value of < 0.05 was considered significant. To ensure the reliability of CBCT data readings, all measurements for 25% of the sample were randomly selected and measured by the same author at a 2-week interval. The results of the paired t-test showed the reliability of readings (p > 0.05). The internal consistency reliability test showed that Cronbach`s alpha was 0.85, which was of high reliability.

## Results

### Comparison of the cephalometric variables at before and after treatment

The cephalometric changes in adults with Class III malocclusion at T0 and T1 time points were shown in Table 1. According to the result of the paired t-test, SNA and SNB were significantly increased (p < 0.05), while ANB, Wits, MP-OP and OP-FH had no statistical differences. IMPA still significantly decreased after orthodontic treatment of distal movement of lower dentition. L6-MPR, L6-MPL, L7-MPR and L7-MPL were statistically increased (more than 7 degrees), which indicated the distal inclination of mandibular molars owing to the mandibular dentition distalization.

**Table 1:** Comparison of the cephalometric variables before and after treatment

	Before Treatment (T0)		After Treatment (T1)		t	p
	Mean	SD	Mean	SD		
SNA (°)	80.46	4.10	82.49	4.62	-2.19	0.042*
SNB (°)	79.78	5.24	81.76	4.80	-2.46	0.024*
ANB (°)	0.68	2.61	0.71	3.00	-0.08	0.934
Wits (mm)	-3.71	3.17	-3.320	2.62	-1.66	0.114
IMPA (°)	90.61	6.87	87.96	6.48	2.37	0.029*
MP-OP (°)	20.58	3.88	19.89	4.51	-1.17	0.256
OP-FH (°)	5.76	5.49	6.20	5.14	1.10	0.285
L6-MPR (°)	101.03	4.59	108.25	5.20	-6.07	< 0.001*
L6-MPL (°)	100.13	6.15	108.85	5.84	-5.95	< 0.001*
L7-MPR (°)	102.04	8.60	109.03	7.09	-6.01	< 0.001*
L7-MPL (°)	97.19	12.16	108.27	7.35	-4.40	< 0.001*

* P < 0.05

### Comparison of the alveolar bone around the mandibular central incisors and canines before and after treatment

According to Table 2, the alveolar bone thickness of the mandibular central incisors on the lingual side decreased significantly on all the 5 levels, while on the labial side only the alveolar bone closed to the crest were absorbed significantly. Although La2-La5 showed no significant differences before and after treatment, the alveolar bone thickness slightly increased after treatment. Similarly, there were significant decreases in the alveolar bone thickness of canines on La1 and lingual side between T0 and T1 (Table 3). La3-La5 were statistically increased after treatment.

**Table 2:** Comparison of the alveolar bone thickness on both labial and lingual side of mandible central incisors before and after treatment

	Before Treatment (T0)		After Treatment (T1)		t	p
	Mean	SD	Mean	SD		
Alveolar bone thickness (labial side of incisors)						
La1	0.42	0.18	0.21	0.20	3.64	0.002*
La2	0.31	0.17	0.26	0.30	0.76	0.458
La3	0.33	0.22	0.44	0.34	-1.34	0.196
La4	0.72	0.40	0.92	0.66	-1.4	0.267
La5	2.58	0.85	2.82	1.12	-0.96	0.347
Alveolar bone thickness (lingual side of incisors)						
Li1	0.38	0.35	0.13	0.21	3.45	0.003*
Li2	0.73	0.48	0.29	0.41	3.63	0.002*
Li3	1.22	0.68	0.62	0.62	3.63	0.002*
Li4	1.96	0.90	1.32	0.91	3.65	0.002*
Li5	3.91	0.95	3.29	1.50	2.45	0.024*

* P < 0.05

**Table 3:** Comparison of the alveolar bone thickness on both labial and lingual side of mandible canines before and after treatment

	Before Treatment (T0)		After Treatment (T1)		t	P
	Mean	SD	Mean	SD		
Alveolar bone thickness (labial side of canines)						
La1	0.48	0.24	0.27	0.22	3.36	0.003*
La2	0.31	0.14	0.29	0.30	0.35	0.732
La3	0.38	0.15	0.73	0.63	-2.59	0.018*
La4	0.93	0.60	1.71	1.04	-3.638	0.002*
La5	3.48	0.88	4.25	1.80	-2.468	0.023*
Alveolar bone thickness (lingual side of canines)						
Li1	0.92	0.38	0.59	0.46	3.560	0.002*
Li2	1.76	0.63	1.39	0.90	2.440	0.025*
Li3	2.39	0.81	2.02	1.09	2.945	0.008*
Li4	3.22	1.07	2.87	1.19	1.834	0.082*
Li5	5.02	0.84	4.67	1.25	1.417	0.173

* P < 0.05

As to the root length, both central incisors (from 10.00 ± 0.75 mm to 9.62 ± 0.98 mm) and canines (from 13.45 ± 1.10 mm to 12.84 ± 1.39 mm) were significantly decreased (p < 0.001). The vertical marginal bone levels of central incisors and canines were significantly reduced after mandibular dentition distalization on both labial and lingual sides. The area of black triangle between the central and lateral incisor was statistically increased from 2.22 ± 0.70 mm2 to 3.89 ± 1.45 mm2. In addition, the area of black triangle between the lateral incisor and canine was increased from 3.12 ± 0.99 mm2 to 4.20 ± 1.34 mm2 (Table 4).

**Table 4:** Comparison of the root length, vertical marginal bone level and black triangle areas before and after treatment

	Before Treatment (T0)		After Treatment (T1)		t	P
	Mean	SD	Mean	SD		
Root length						
Central incisor (mm)	10.00	0.75	9.62	0.98	3.71	0.001*
Canine (mm)	13.45	1.10	12.84	1.39	4.04	0.001*
Vertical marginal bone level						
Labial side of incisor (mm)	1.12	0.38	1.84	0.67	-4.92	< 0.001*
Lingual side of incisor (mm)	1.42	0.51	2.68	1.74	-3.07	0.006*
Labial side of canine (mm)	1.60	0.42	2.88	2.33	-2.53	0.021*
Lingual side of canine (mm)	1.41	0.43	2.14	0.72	-3.79	0.001*
Black triangle area						
S12 (mm^2^)	2.22	0.70	3.89	1.45	-5.82	< 0.001*
S23(mm^2^)	3.12	0.99	4.20	1.34	-4.68	< 0.001*

* P < 0.05

Comparisons of total pharyngeal airway volume showed no significant differences before and after treatment, the same as palatopharyngeal airway volume, glossopharyngeal airway volume, laryngopharyngeal airway volume and minimum axial area. However, the above numerical values declined slightly after treatment based on the mean values in Table 5. The minimum axial area showed a nonsignificant decrease of 6.85 mm2 (p > 0.05).

**Table 5:** Comparison of the pharyngeal airway volumes and minimum axial area before and after treatment

	Before Treatment (T0)		After Treatment (T1)		t	P
	Mean	SD	Mean	SD		
Palatopharyngeal airway volume (mm^3^)	7632.65	2808.87	7110.35	2110.25	1.57	0.132
Glossopharyngeal airway volume (mm^3^)	5293.95	2504.89	4857.35	1668.92	1.50	0.150
Laryngopharyngeal airway volume (mm^3^)	2957.65	1264.98	2756.05	1268.19	2.03	0.057
Pharyngeal airway volume (mm^3^)	15884.25	5731.97	14723.75	4011.37	1.93	0.069
Minimum axial area (mm^2^)	190.65	87.50	183.80	70.54	0.44	0.665

* P < 0.05

## Discussion

The pursuits of orthodontic treatment are not only the coordination of stomatognathic system, stability of dentofacial structures and aestetic appearance but also the normal functioning of periodontium, airway and temporomandibular joints [17]. Especially in patients with Class III malocclusion, the alveolar bone of mandibular anterior teeth is thinner than that in patients with other kinds of malocclusions [18]. Hence, the teeth movement and bone remodeling of Class III patients are restricted by the anatomical boundary [18, 19]. Before the orthodontic treatment, periodontal problems should be carefully assessed during clinical examination. Furthermore, prudent orthodontic treatment should be performed to avoid iatrogenic periodontal injury or aggravating the periodontal deficiencies.

CBCT images were taken before and after orthodontic treatment for the evaluation described above. For the concern of radiation exposure regarding the dose of CBCT scans, it han been reported that the radiation exposure of taking one CBCT scan is about 0.25mSV. According to the International Radiation Protection Association (IRPA), the radiation exposure received by each person every year is about 2.5mSv [20]. And the time interval between pre-treatment and post-treatment scans was relatively long, at least one year apart. Hence, the CBCT scans were safe for the patients in this study. Furthermore, one of the most accurate methods to evaluate the alveolar bone in three dimensions is the use of CBCT images. It was reported that there was a high degree of accuracy in measurements of alveolar bone height and thickness between CBCT and direct measurements [21]. And it could eliminate the confounding factor of the soft-tissue variations.

According to the cephalometric analysis in Table 1, ANB and Wits had no statistical differences before and after mandibular dentition distalization. The lingual inclination of mandibular incisors was significantly decreased from 90.61 ± 6.87° to 87.96 ± 6.87° (p < 0.05). Although the treatment goal was an overall distalization of the incisors and molars, the result showed that the lingual inclination of mandibular incisors was inevitably to a certain extent, which was similar to the previous study of 4.8° [22]. In other words, the incisor retraction was a combined movement that included both translation and tipping. The angle between the mandibular first/second molar and the mandibular plane on both left and right sides increased statistically after orthodontic treatment. It was most likely due to the need for space for distalization, direction of force application, and anchorage control during distal movement of the lower dentition. The previous study [22] indicated that distalization at the crown levels of mandibular first molar was 1.8 mm, 0.6 mm at the root levels with distal tipping of 5.4° using the buccal miniscrew. Similarly, Sugawara found that distalization at the the crown levels of mandibular first molar was 3.5 mm and a distal tipping ratio of 46.3% with the use of anchor plate [23]. Furthermore, there was no significant relationship between the amount of relapse and the tipping ratio or the amount of mandibular distalization.

Regarding the comparison of the alveolar bone thickness around the mandibular incisors and canines before and after treatment, the alveolar bone thickness of the mandibular central incisors on the lingual side decreased significantly on all the 5 levels, while on the labial side only the alveolar bone closed to the crest were absorbed significantly. It indicated that the alveolar bone closed to the crest were more sensitive to the stress concentration from the overall distal movement of the lower incisor [24]. Besides, there were significant decreases in the alveolar bone thickness of canines on La1 and most parts of lingual side between T0 and T1 (p < 0.05). The marginal thin layer of alveolar bone on the lingual side was more possible to be absorbed during tooth movement [10], which suggested that careful periodontal examination before treatment was of great importance to evaluate the feasibility of distalization.

From the excess analysis of cervical area around mandibular incisors,the marginal bone level of incisors was increased from 1.12 ± 0.38 mm to 1.84 ± 0.67 mm on the labial side. The deterioration was smaller than that on the lingual side, which was increased from 1.42 ± 0.51 mm to 2.68 ± 1.74 mm (Table 4). Similar to the incisors, the absorption of the cervical bone around canines was increased significantly (p < 0.05). Flavio [25] found that alveolar bone width was decreased on average from 17 to 25% and the height of the ridge was decreased 2–10 mm after orthodontic space opening. Similarly, Garlock [26] evaluated marginal alveolar bone height of mandibular incisors and the result showed that labial and lingual vertical bone losses on average were 1.16 ± 2.26 and 1.33 ± 2.50 mm. The incisor inclination was not related to the changes in alveolar bone height, and tooth translation had a greater tendency to result in the loss of alveolar bone. Besides, the enlargement of the black triangle in the anterior teeth visually revealed the vertical bone loss. This suggested once the periodontal condition tends to be worse, periodontal assisted orthodontic treatment such as partial corticotomy with bone graft, periodontally accelerated osteogenic orthodontic (PAOO) [27] should be considered in time.

The statistically significant differences found between T0 and T1 time point was the root length of central incisors, which decreased 0.38 mm along with canines 0.61 mm after mandibular distalization, which was in line with previous studies [28]. Lombardo [29] showed that the root resorption of lower incisor after orthodontic treatment with extraction was 1.00 mm and 0.73 mm without extraction, and there was significant difference between them. The root resorption that we found was less than the aforementioned studies. It was reported that the type and magnitude of orthodontic force, treatment duration, age, and gender have been associated with the root resorption. Significantly related risk factors were prolonged treatment duration and treatment with extraction [30]. Therefore, the root length should be monitored in patients with orthodontic treatment longer than six months. The mechanism of root resorption was most likely related to the application of an orthodontic force. On the pressure side, not only the bone thickness, but also the root length was reduced. According to the literature, when root resorption was within the limit of 2 mm, there was no negative effect on dental health. The distal movement of the lower dentition is relatively safe for the root of anterior teeth in correcting patients with mild to moderate Class III malocclusion. However, orthodontic treatment with long movements of incisors or extraction and long treatment duration should be taken more care of the greater risk of root resorption [31].

Regarding the pharyngeal airway space analysis, Irani [32] reported significant decreases in all pharyngeal airway volumes after isolated mandibular setback surgery, which showed that posteriorly displaced tongue could narrow the retrolingual area and decrease the airway space. Karaman [13] stated that premolar extraction during orthodontic treatment did not affect the airway dimensions much. Prak [33] revealed that the amount of maxillary distalization in adults had an inverse correlation with the changes in airway. It meant that the larger the distalization of the maxillary dentition, the smaller the airway volume displayed. Also, there were no relations in the nonextraction group. Few studies have been performed on the effects of mandibular dentition distalization on the upper airway. Our study showed that no significant changes were found in the palatopharyngeal airway volume, glossopharyngeal airway volume and laryngopharyngeal airway volume. It suggested that the amount of distalization in mandible was not large enough to bring about a change in the airway space. However, all the values declined slightly after treatment. The total pharyngeal airway showed a non-significant decrease of 1160.5 cm3 in airway volume, while the minimum axial area decreased by 6.85 mm2. The reason why more attention should be paid to airway health during orthodontic treatment was that a reduced arch depth in the sagittal plane may result in decreased oral cavity volume and posterior displacement of the tongue and soft palate, which would lead to possible aggravation of OSA [34]. In addition, there was a significant association between OSA and periodontitis, which was caused by the hypoxia owing to OSA [35]. Accordingly, airway analyses should be taken into account during treatment planning, especially for those who had an OSA tendency. Attention should also be paied to treatment feedback and appropriate solutions such as changing the orthodontic plans or seeking the help of surgical operations.

## Conclusions

Mandibular molar teeth inclined distally to provide more space required by distalization. Mandibular dentition distalization could result in the loss of alveolar bone around anterior teeth in Class III malocclusion, especially for the cervical marginal bone. Pharyngeal airway dimensions were not affected to a high degree after distalization. Hence, periodontal condition and airway health should be taken into prudent consideration during the whole course of orthodontic treatment.

## Data Availability

The datasets generated and analyzed during the current study are not publicly available due to the informed consent but are available from the corresponding author on reasonable request.
